# *EZH2* expression in hepatocellular carcinoma and its relationship with circadian rhythm-related genes

**DOI:** 10.1038/s41598-025-26175-x

**Published:** 2025-11-26

**Authors:** Xingyue Wang, Miaolong He, Limian Zhou, Wei Chen

**Affiliations:** 1 Department of Nuclear Medicine, The Second Affiliated Hospital of Nanjing Medical University, Nanjing, 210003 Jiangsu China; 2 Department of Gynecology, Binzhou Medical University Hospital, Binzhou, 256600 China; 3 Department of General Surgery, The First People’s Hospital of Wu Hu, No. 1 Chi zhu Shandong Road, Jiujiang District, Wuhu City, 241000 Anhui Province China

**Keywords:** EZH2, Biomarker, Circadian rhythm, Hepatocellular carcinoma, Prognosis, Cancer, Genetics, Immunology, Molecular biology

## Abstract

Hepatocellular carcinoma (HCC) is the most prevalent liver malignancy, with increasing incidence and high mortality, primarily associated with hepatitis virus infection and cirrhosis. Herein, we explored the association between enhancer of zeste homolog 2 (*EZH2*) expression and HCC progression, prognosis, and circadian rhythm-related genes. Differentially expressed mRNAs in HCC were identified from The Cancer Genome Atlas and Gene Expression Omnibus databases and intersected with circadian rhythm-related genes. Core genes were screened using protein–protein interaction networks, least absolute shrinkage and selection operator regression, and Cox regression analysis. Functional enrichment and immune infiltration analyses were conducted using gene set variation analysis, single-sample gene set enrichment analysis, and MethSurv. Experimental validation included western blotting, small interfering RNA knockdown, reverse transcription quantitative polymerase chain reaction, and functional assays. *EZH2* was overexpressed in HCC and was associated with advanced tumor stage, poor prognosis, Th2 and dendritic cell infiltration, and promoter hypermethylation. *EZH2* knockdown inhibited proliferation, induced DNA damage, and downregulated *CLOCK* and *CRY1* expression. A prognostic nomogram integrating *EZH2* expression with clinicopathological parameters demonstrated strong predictive performance. Methylation analysis identified six CpG sites significantly associated with survival. In conclusion, EZH2 is a key regulator of HCC progression and potential prognostic biomarker and therapeutic target.

**Supplementary Information:**

The online version contains supplementary material available at 10.1038/s41598-025-26175-x.

## Introduction

Hepatocellular carcinoma (HCC) is the most prevalent form of liver malignancy worldwide, with a significant increase in its incidence and mortality rates. The World Health Organization reported that liver cancer was responsible for nearly 830,000 fatalities in 2020, making it the third most common cause of cancer-related deaths globally^1^. The increasing prevalence of HCC is closely associated with risk factors, including chronic hepatitis B and C infections, liver cirrhosis, and metabolic disorders such as nonalcoholic fatty liver disease^2^. Current treatment strategies—including surgical resection, liver transplantation, and systemic therapies—often show limited efficacy, particularly in the advanced phases of the disease, underscoring the urgent need for novel therapeutic targets and prognostic biomarkers^3^.

Recent studies have demonstrated a close association between circadian rhythm disruption and the initiation and progression of HCC. Circadian rhythm, an intrinsic and highly sophisticated timekeeping system within various organisms, regulates numerous physiological processes essential for life, including the cell cycle, metabolic mechanisms, and DNA repair^4^. Disruption of circadian rhythms has been implicated in metabolic disorders and tumor development and progression by influencing mechanisms such as cell proliferation, apoptosis, and DNA repair^5^. Domestic and international studies have revealed abnormal expression of multiple circadian rhythm-related genes (e.g., *CLOCK*, *ARNTL*, *PER1*, *CRY1*) in HCC and their correlation with patient prognosis^6,7^. For instance, the aberrant expression of the *CLOCK* gene has been linked to enhanced invasiveness and metastatic potential in HCC^5^, whereas reduced expression of *PER1* is correlated with unfavorable outcomes in patients with HCC^8^.

Recently, the significance of epigenetic regulation in HCC has attracted increasing attention. enhancer of zeste homolog *2* (*EZH2*), the central catalytic component of polycomb prepressive complex 2 (*PRC2*), plays a pivotal role in transcriptional silencing, cell cycle regulation, and the maintenance of stem cell pluripotency by catalyzing histone H3K27 trimethylation (H3K27me3)^9^. Elevated *EZH2* expression is associated with poor prognosis in various cancers, including HCC, suggesting its potential as a prognostic biomarker^10^, the interaction between *EZH2* expression and circadian rhythm-related genes has been emphasized, as disturbances in circadian rhythms can influence tumor development and patient outcomes^11^. The association between *EZH2* and circadian genes, including *CLOCK* and *BMAL1*, could shed light on the molecular mechanisms that drive HCC progression and underscore the significance of circadian regulation in cancer biology.

This study aimed to examine the relationship between the expression of genes associated with circadian rhythms and HCC, with particular emphasis on the central gene *EZH2*. This gene is associated with tumor development and may act as a prognostic indicator in individuals diagnosed with HCC. This study integrated large-scale genomic data with advanced statistical methodologies to shed light on the molecular processes that underlie HCC, revealing *EZH2* as a key factor influencing patient prognosis. Accordingly, the findings may contribute to the development of targeted therapeutic approaches and enhance the accuracy of prognostic evaluations in patients with HCC.

## Results

### Screening for core genes between differentially expressed genes (DEG) and circadian rhythm-related genes

**Fig. 1:**
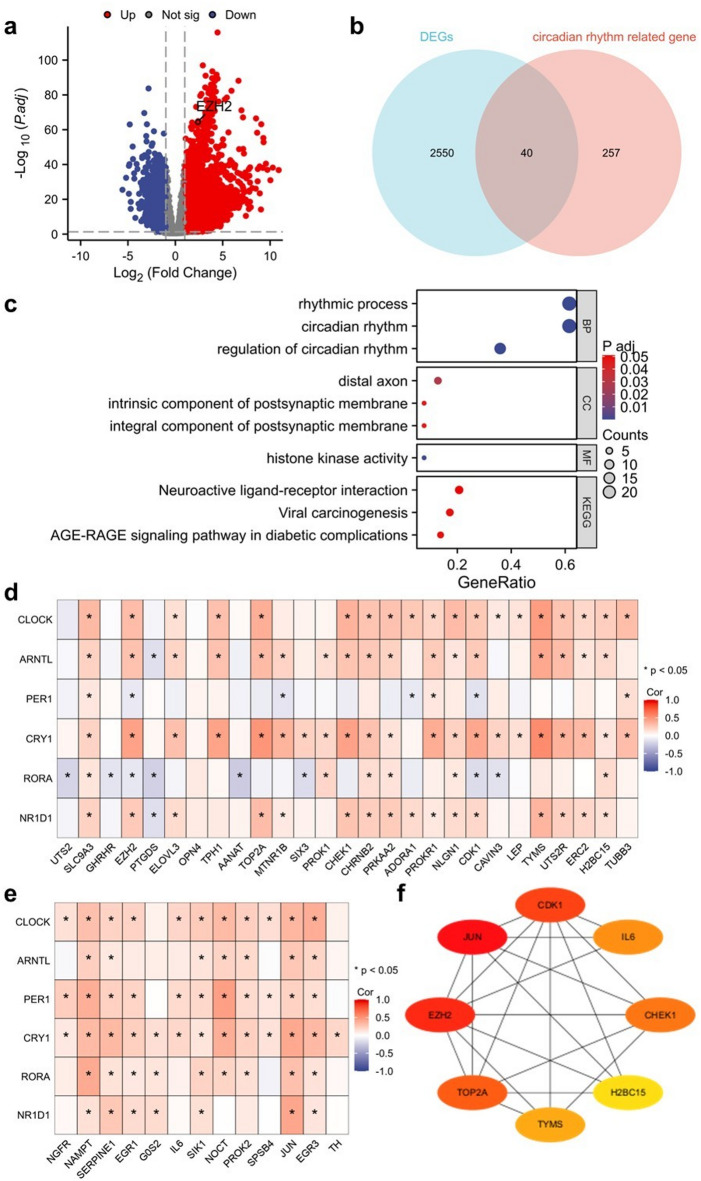
Distributions of mRNA in HCC and identification of DEGs associated with circadian rhythm-related genes. (**a**) Volcano plot of 2588 DEG. The red and blue dots represent the upregulated and downregulated genes, respectively. (**b**) Venn diagram of circadian rhythm-related genes and DEGs of HCC patients. The blue circle represents HCC-related DEGs, and the pink circle represents circadian rhythm-related genes. (**c**) GO and KEGG enrichment analyses of 27 upregulated genes and 13 downregulated genes. (**d**) Spearman correlation analysis between 27 upregulated genes and circadian rhythm-related genes* (*p* < 0.05). (**e**) Spearman correlation analysis between the 13 downregulated genes and circadian rhythm-related genes * (*p* < 0.05). (**f**) Hub genes in 27 upregulated genes and 13 downregulated genes.

Comparative analysis of HCC versus normal liver tissues revealed 2590 DEGs with 2036 genes upregulated and 554 downregulated genes, as illustrated by the volcano plot in Fig. 1a. We screened circadian rhythm-associated genes from the Molecular Signatures Database (MSigDB) to delve deeper into the circadian rhythm–HCC nexus. We used Venn diagrams to precisely isolate genes concurrently dysregulated in HCC and circadian control. Intersection analysis of DEGs with those associated with circadian rhythms revealed 40 differentially expressed circadian rhythm-related genes (DECRGs) (Fig. 1b). The 40 DECRGs were then subjected to Gene Ontology (GO) and Kyoto Encyclopedia of Genes and Genomes (KEGG) (GOKEGG) enrichment analyses, including molecular function (MF), components of cells (CC), and biological processes (BP) categories. The results indicated that BP closely tied to circadian rhythms, including rhythmic processes, circadian rhythms, and their regulation, were notably enriched (Fig. 1c). The CC analysis highlighted the enrichment of DECRGs at specific neuronal loci, enhancing our understanding of the HCC cell microenvironment. MF analysis underscored histone kinase activity as the primary function among the 40 overlapping genes, suggesting a pivotal role for epigenetic modifications in regulating the circadian rhythm and tumorigenesis in HCC (Fig. 1c). KEGG pathway enrichment analysis further revealed pivotal links to neuroactive ligand–receptor crosstalk, viral-induced carcinogenesis, and the AGE-RAGE signaling pathway in diabetic complications (Fig. 1c). These findings implicate multifactorial mechanisms in HCC progression, including neural, viral, and metabolic disruptions, and provide a molecular framework for its complex etiology. Several core circadian genes are particularly important, including *CLOCK*, *ARNTL*, *PER1*, *CRY1*, *RORA*, and *NR1D1*^6^. Correlation analysis of selected upregulated (Fig. 1d) and downregulated (Fig. 1e) overlapping genes revealed associations with *CLOCK* and *CRY1*, among others. Subsequently, a gene–gene interaction network was constructed based on the 40 DECRGs to explore the central hub genes. Network analysis identified eight key hub genes regulating this complex network (Fig. 1f).

**Fig. 2:**
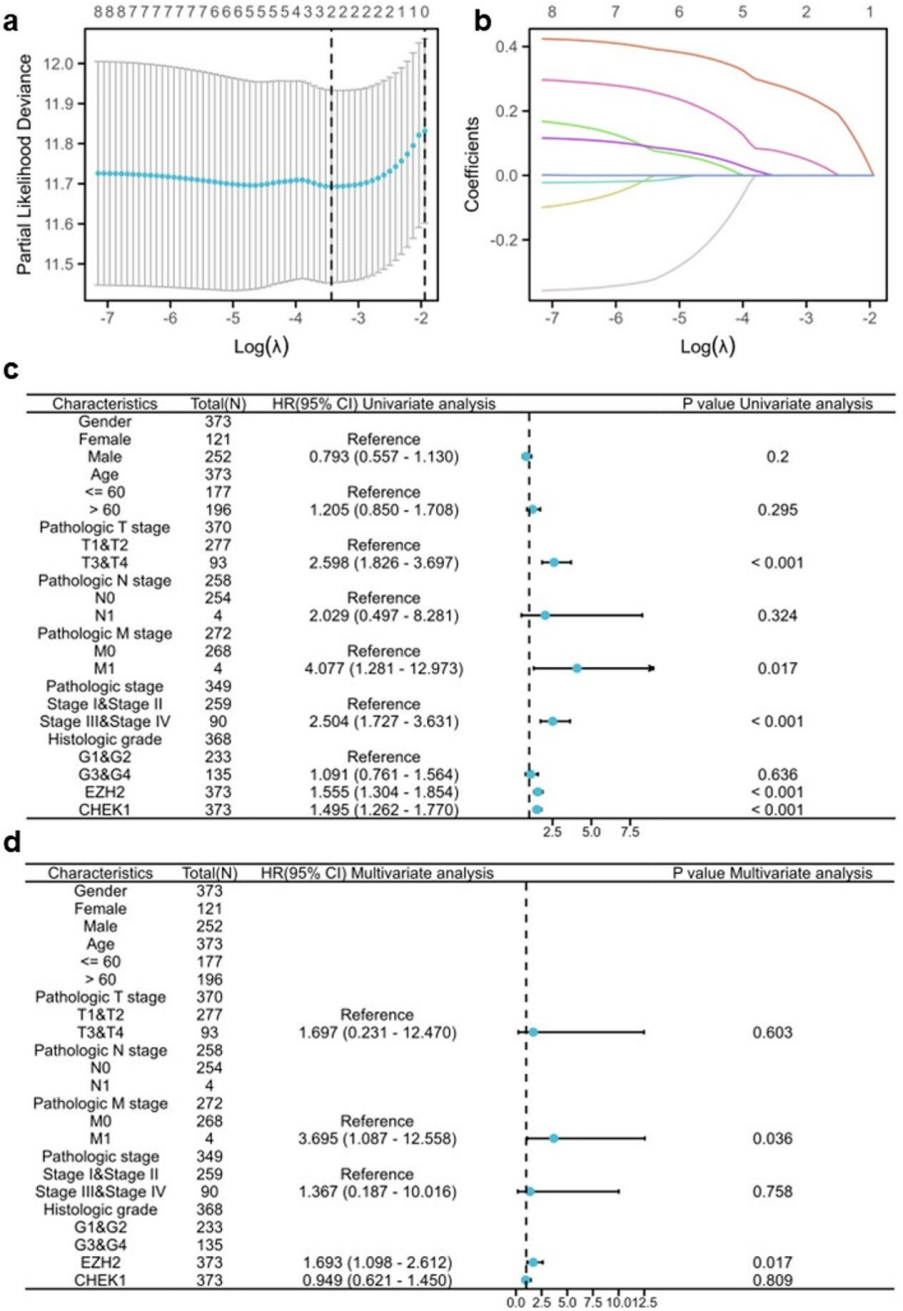
Prognostic LASSO regression and cox analysis of *CHEK1* and *EZH2* expression. (**a**) Prognostic LASSO coefficient screening for the eight hub genes. (**b**) Prognostic LASSO variable trajectories for the eight hub genes. (**c**) Univariate Cox analysis of *CHEK1* and *EZH2* expression and other clinicopathological variables. (**d**) Multivariate Cox analysis of *CHEK1* and *EZH2* expression and other clinicopathological variables.

These eight hub genes were further filtered using least absolute shrinkage and selection operator (LASSO) regression analysis, which identified two genes that emerged as the most significant predictors (Fig. 2a-b). Moreover, univariate and multivariate Cox regression analyses were performed to explore the association between the expression levels of the two genes and the overall survival (OS) duration of patients within the HCC cohort, which included clinical variables. (Fig. 2c, d). These results suggested that *EZH2* is an independent risk factor for HCC.

### High expression of EZH2 in HCC

**Fig. 3:**
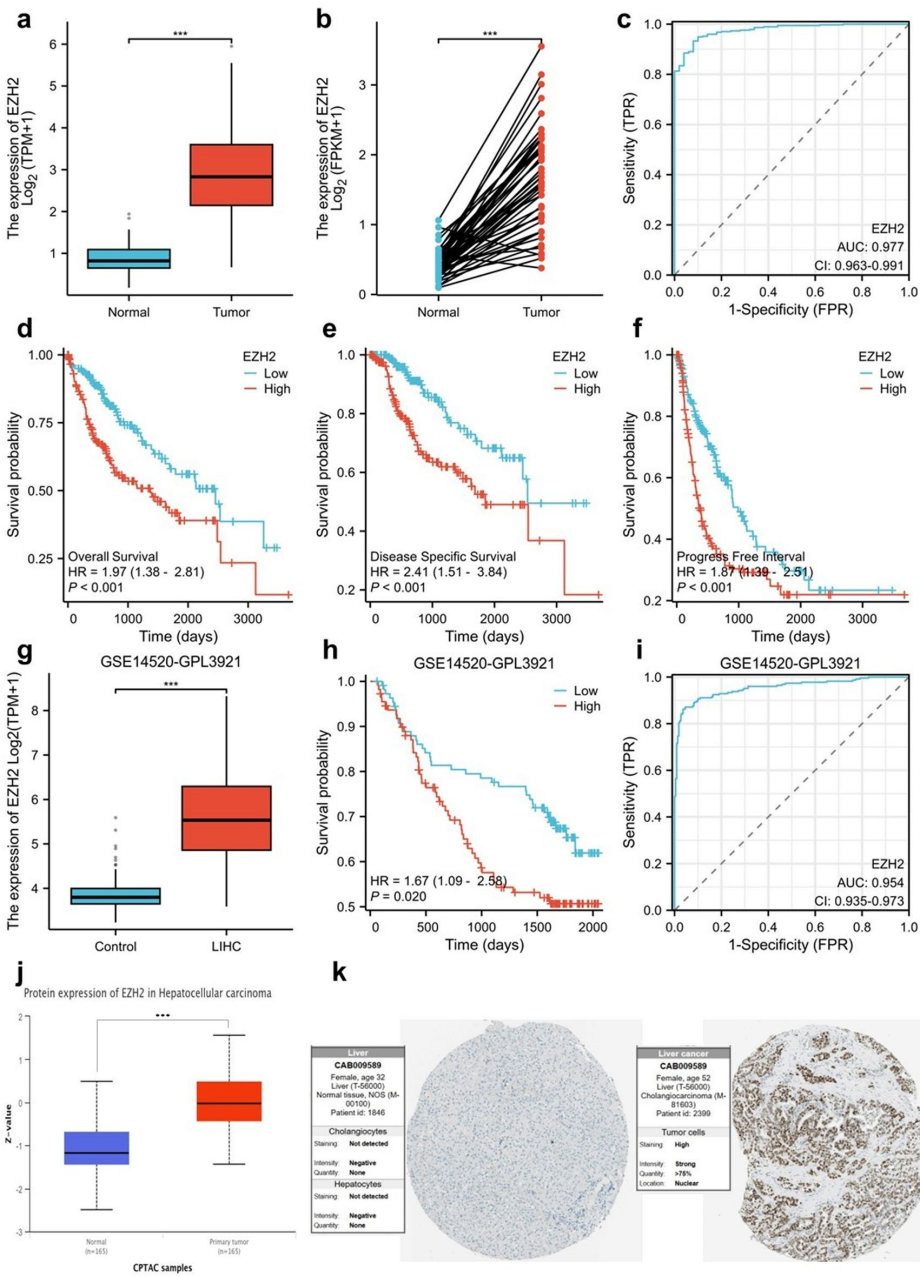
Expression and prognostic value of *EZH2* in HCC. (**a**–**b**) The expression of *EZH2* in normal versus tumor tissues from TCGA cohort. (**c**) ROC curve assessing the diagnostic value of *EZH2* in HCC patients (TCGA). (**d–f**) Kaplan–Meier survival curves comparing the high and low expression of *EZH2* with regards to OS (D), DSS (E), and PFI (F) in TCGA. (**g**) Expression of *EZH2* in normal and tumor tissues from the GEO dataset. (**h**) Kaplan–Meier survival curve comparing the high and low expression of *EZH2* expression for OS in GEO. (**i**) ROC curve evaluating the diagnostic performance of *EZH2* in HCC patients in (GEO). (**j**) *EZH2* expression in normal versus tumor tissues using UALCAN. (**k**) Representative immunohistochemical staining for *EZH2* in normal liver and HCC tissues from the HPA(used with permission).

Through a detailed examination of gene expression datasets sourced from The Cancer Genome Atlas (TCGA) database, we identified a significant increase in the expression levels of the *EZH2* within HCC tissues compared with normal liver tissues (Figs. 3a, b). These findings offer preliminary evidence for the involvement of *EZH2* in HCC progression. We calculated an area under the curve (AUC) of 0.977, indicating strong diagnostic potential for *EZH2* in distinguishing HCC from normal tissues (Fig. 3c). Patients diagnosed with HCC were categorized into cohorts based on the levels of *EZH2* expression, revealing significantly prolonged OS, disease-specific survival (DSS), and progression-free interval (PFI) in the low-*EZH2* group (*p* < 0.001), with notably extended survival times (Fig. 3d–f). This finding support the potential of *EZH2* as a prognostic biomarker, further elucidating the *EZH2*–clinical outcome correlation. In addition, we used the Gene Expression Omnibus (GEO) database and UALCAN to validate our findings. The results were consistent with those from TCGA database, showing that *EZH2* expression was elevated in cancer tissues relative to normal tissues (Fig. 3g, j), with an AUC of 0.954 (Fig. 3i), and high expression of *EZH2* was associated with poor prognosis (Fig. 3h). Immunohistochemical staining images of normal liver and tumor tissues were obtained from the Human Protein Atlas (HPA) database. The images indicated that the expression level of *EZH2* in HCC tissues was significantly higher than that in normal tissues (Fig. 3k).

### Correlation between EZH2 expression levels and clinicopathological characteristics of patients with HCC

Employing TCGA database, we analyzed the correlation between *EZH2* expression and diverse clinical parameters in patients with HCC (Table 1). We specifically examined the association between *EZH2* expression and 10 key clinicopathological features of HCC, including patient demographics (age and sex), tumor stage (T, N, M), pathological stage, presence of residual tumor, histological grading, and OS events (Supplementary Fig. 2). Our findings revealed a statistically significant correlation between elevated *EZH2* expression and advanced T stage (Supplementary Fig. 2d), higher pathological stage (Supplementary Fig. 2f), poorer histological grade (Supplementary Fig. 2H), and reduced OS (Supplementary Fig. 2i), all with *p* < 0.001. These findings indicate marked upregulation of *EZH2* in HCC, which correlates with multiple adverse clinical parameters, reinforcing its potential utility as a prognostic marker.

**Table 1:** Relationship between *EZH2* expression and clinicopathological features in TCGA database.

Variable	Low expression of EZH2	High expression of EZH2	*p*-value
n	187	187	
Gender, n (%)			0.224
Female	55 (14.7%)	66 (17.6%)	
Male	132 (35.3%)	121 (32.4%)	
Age, n (%)			0.043
≤ 60	79 (21.2%)	98 (26.3%)	
> 60	108 (29%)	88 (23.6%)	
Pathologic T stage, n (%)			0.088
T1&T2	145 (39.1%)	133 (35.8%)	
T3&T4	39 (10.5%)	54 (14.6%)	
Pathologic N stage, n (%)			0.670
N0	123 (47.7%)	131 (50.8%)	
N1	1 (0.4%)	3 (1.2%)	
Pathologic M stage, n (%)			0.583
M0	130 (47.8%)	138 (50.7%)	
M1	3 (1.1%)	1 (0.4%)	
Histologic grade, n (%)			0.000
G1&G2	138 (37.4%)	95 (25.7%)	
G3&G4	47 (12.7%)	89 (24.1%)	
Pathologic stage, n (%)			0.050
Stage I & Stage II	138 (39.4%)	122 (34.9%)	
Stage III & Stage IV	37 (10.6%)	53 (15.1%)	

### Construction and validation of a prognostic and diagnostic model

**Fig. 4:**
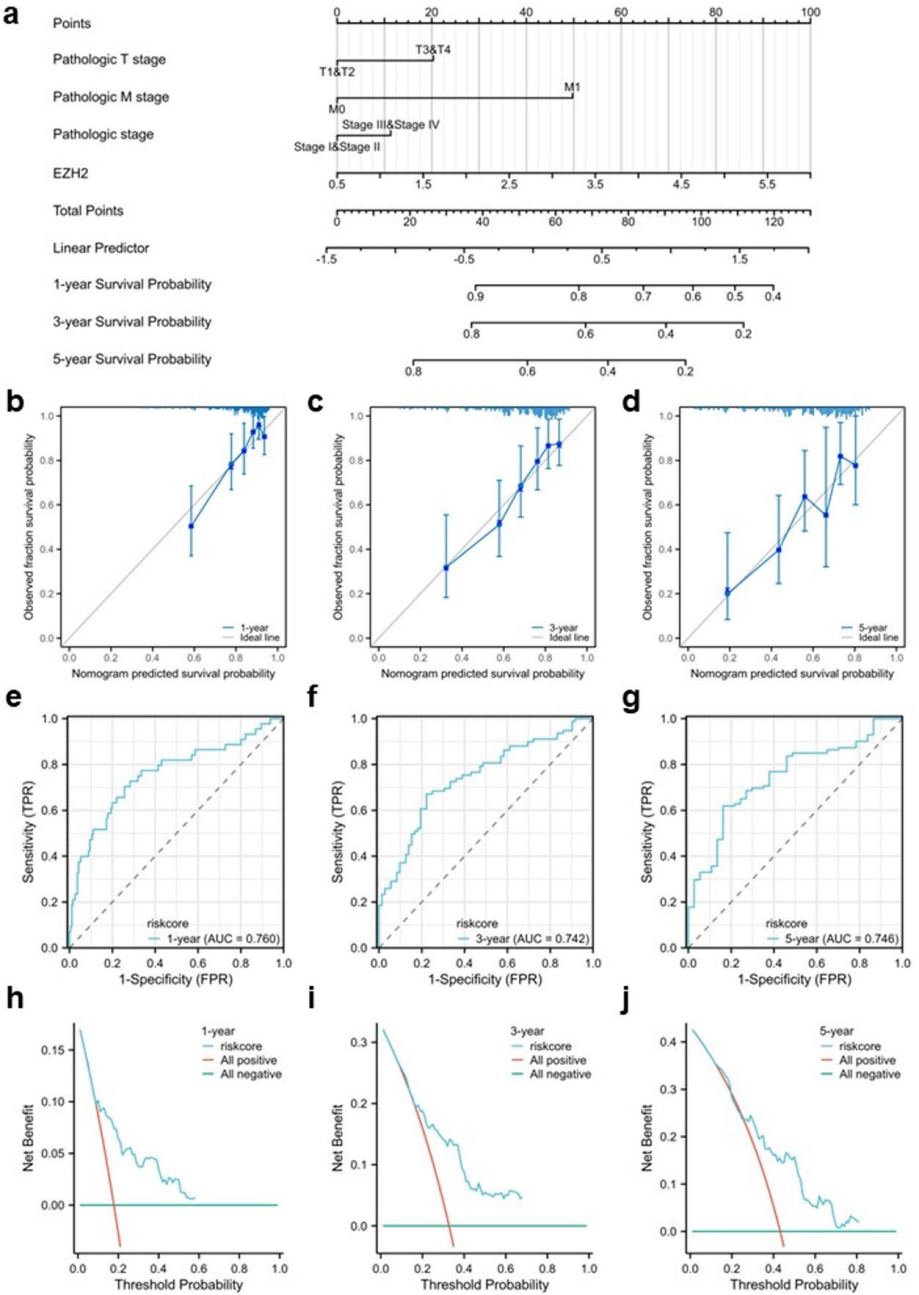
Predictive performance of *EZH2* for OS in HCC. (**a**) Nomogram predicting 1-, 3-, and 5-year OS based on *EZH2* expression and clinical variables. (**b**–**d**) Calibration plots for 1-, 3-, and 5-year OS used to validate the nomogram model. (**e–g**) Time-dependent ROC curves for 1-, 3-, and 5-year OS. (**h–j**) DCA for 1-, 3-, and 5-year OS.

We established prognostic models to improve clinical decision-making and advance personalized medicine by selecting risk factors derived from the Cox regression analysis. A nomogram was constructed based on key predictors of OS in HCC, including T stage, M stage, pathological stage, and *EZH2* expression, to further validate the prognostic significance of *EZH2* on survival rates at 1, 3, and 5 years. The cumulative score, derived by summing the scores assigned to each prognostic factor, was used to predict the OS of patients with HCC. The nomogram revealed a higher total score associated with a poorer prognosis (Fig. 4a). Furthermore, calibration curves were utilized to evaluate the alignment between the predicted probabilities from the nomogram at different time intervals and the actual survival probabilities, indicating that the model could provide an accurate estimation of survival probabilities for 1-, 3-, and 5-year survival rates (Fig. 4b–d). Receiver operating characteristic (ROC) curve analysis showed that the AUC values for time-dependent ROC at 1, 3, and 5 years were 0.760, 0.742, and 0.746, respectively. These results suggest that the prognostic model demonstrated a strong predictive capability (Fig. 4e–g). Decision curve analysis (DCA) is a statistical method used to evaluate the clinical utility of predictive models in practical applications by comparing the net benefit of different models across various threshold probabilities, assisting physicians and researchers. The DCA revealed that the model provided valuable guidance for clinical decision-making within this threshold range (Fig. 4h–j).

### Functional enrichment analysis of EZH2 in patients with HCC

We analyzed genes co-expressed with *EZH2* in the TCGA HCC dataset. A total of 8301 genes were identified based on the criteria of |Spearman’s correlation coefficient| > 0.3 and *p* < 0.05. The top 30 positively correlated genes are shown in Supplementary Fig. 3a. Functional annotations of these co-expressed genes were explored using GO terms and KEGG pathway analysis. Bubble plots illustrate the top three enriched categories of BP, CC, MF, and KEGG pathways. GO analysis revealed that these genes were predominantly involved in organelle fission, nuclear division, chromosome segregation, various catalytic activities, ATP-dependent mechanisms, and single-stranded DNA helicase activity (Supplementary Fig. 3b). The KEGG pathway analysis highlighted the significant roles of these genes in regulating the cell cycle, DNA replication, and Fanconi anemia pathways (Supplementary Fig. 3b).

Moreover, gene set variation analysis (GSVA) was conducted to explore the potential impact of *EZH2* expression on the development and progression of HCC. The findings revealed that genes in the high- and low-expression cohorts were predominantly enriched in an array of biological pathways such as E2F targets, G2M checkpoint, DNA repair, PI3K/AKT/MTOR signaling, mitotic spindle, spermatogenesis, and MYC targets v1 (Supplementary Fig. 4). These results suggest that genes related to the circadian rhythm may play a significant role in modulating the processes of DNA damage and repair, DNA replication, and the cell cycle.

### EZH2 expression is significantly correlated with immune infiltration

Our investigation explored the relationship between *EZH2* expression and immune cell infiltration in tumors using both single-sample gene enrichment analysis (ssGSEA) and CIBERSORT algorithms. The analysis highlighted the three most significant correlations between *EZH2* and Th2 cells (*r* = 0.662, *p* < 0.01), Th17 cells (*r* = − 0.417, *p* < 0.01), and dendritic cells (DCs) (*r* = − 0.404, *p* < 0.01), as determined by the ssGSEA algorithm (Supplementary Fig. 5a). Concurrently, using the CIBERSORT algorithm, our findings revealed that the three most notable associations were between *EZH2* and follicular helper T cells (*r* = 0.269, *p* < 0.01), M0 macrophages (*r* = 0.239, *p* < 0.01), and activated CD4^+^ memory T cells (*r* = 0.235, *p* < 0.01) (Supplementary Fig. 5b). We further assessed immune cell enrichment scores in high- and low- *EZH2* expression groups using both ssGSEA and CIBERSORT. According to the ssGSEA method, the high-expression group displayed increased enrichment scores in four categories of immune cells, whereas the low-expression group demonstrated higher scores in eight categories of immune cells (Supplementary Fig. 5c). In contrast, the CIBERSORT results showed that the high-expression group had elevated enrichment scores across five immune cell types, whereas the low-expression group had increased scores in two immune cell types (Supplementary Fig. 5d).

### EZH2 gene mutation and methylation

**Fig. 5:**
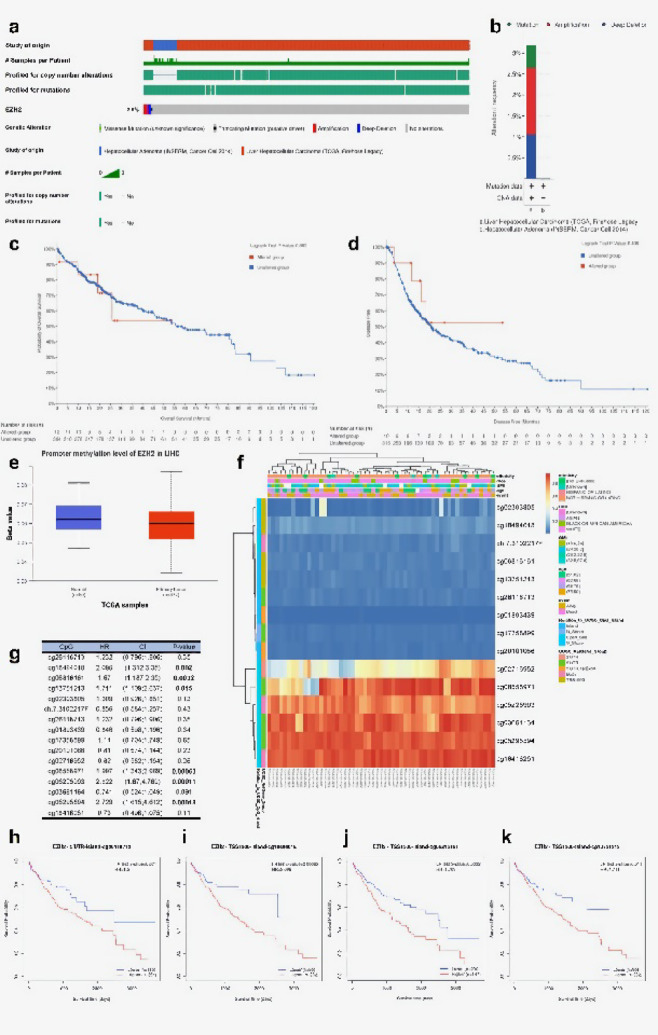
Mutations and DNA methylation levels of *EZH2* and their associations with HCC prognosis. (**a**, **b**) OncoPrint plots showing *EZH2* mutation profiles from the cBioPortal database. (**c**, **d**) Kaplan-Meier survival curves evaluating the association between *EZH2* mutation status and OS (C) and DFS (D) in HCC. (**e**) *EZH2* methylation levels in HCC compared to normal tissue, retrieved form the UALCAN database. (**f**) Correlation between *EZH2* mRNA expression level and methylation level form the MethSurv database. (**g**) Association between *EZH2* methylation levels and HCC prognosis. (**h–k**) Kaplan–Meier survival curves showing the prognostic impact of methylation at specific *EZH2* CpG sites: (**h**) cg26118713, (**i**) cg18494018, (**j**) cg06816161, (**k**) cg13751213.

Given that genetic mutations are a primary factor contributing to the onset of cancer^12^, we examined *EZH2* mutations and copy number variations (CNVs) using two datasets on HCC from cBioPortal: INSERM, Nat Genet 2015 (*n* = 243) and TCGA, Firehose Legacy, (*n* = 379). Our findings indicated that *EZH2* exhibited missense mutations, truncating mutations, amplifications, and deep deletions at a rate of 2.9% (Fig. 5a, b). Irregular DNA methylation plays a crucial role in the initial stages of HCC progression^13,14^. Nevertheless, our investigation determined that mutations in *EZH2* did not correlate with variations in OS (*p* = 0.803) or DFS (*p* = 0.409) (Fig. 5c, d). Further assessments using the UALCAN database indicated that the elevated DNA methylation levels of *EZH2* in the promoter region of HCC tissues compared with normal liver tissues were statistically significant (*p* < 0.01) (Fig. 5e). Furthermore, utilizing the MethSurv database facilitated a more nuanced exploration of the DNA methylation levels of *EZH2* and the prognostic significance of its associated CpG islands. The outcomes revealed that most CpG sites were hypomethylated (Fig. 5f). Importantly, six CpG sites displayed methylation levels associated with HCC prognosis (Fig. 5g). In particular, hypermethylation of cg18494018, cg06816161, and cg13751213 correlated with a worse prognosis (Fig. 5h–k).

### Correlation of EZH2 expression with circadian rhythm in HCC

**Fig. 6:**
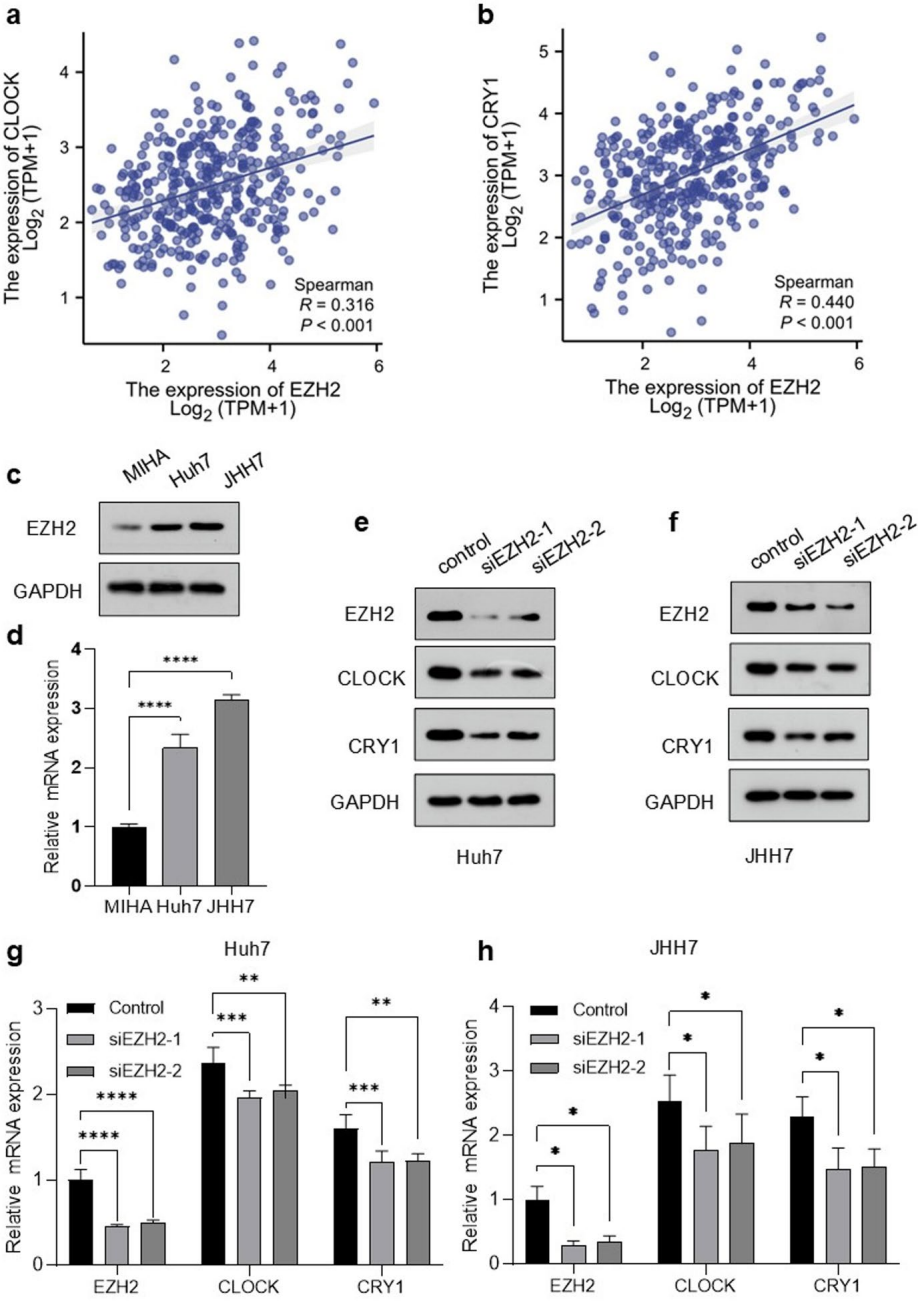
Effect of *EZH2* knockdown on the expression of circadian rhythm-related genes in HCC cells. JHH7 and Huh7 cells were transfected with control or EZH2-targeting siRNAs. (**a**, **b**) Scatter plots were plotted showing the correlations between *EZH2* and expression of circadian rhythm-related genes, including *CLOCK*, and *CRY1*. (**c**) Protein expression of EZH2 in MIHA, Huh7, and JHH7. (**d**) mRNA expression of *EZH2* in MIHA, Huh7, and JHH7. (**e**, **f**) Protein levels of EZH2, CLOCK, and CRY1 as measured by western blotting. (**g**, **h**) mRNA levels of *EZH2*,* CLOCK*, and *CRY1* assessed by real-time quantitative PCR. *p* < 0.05, *p* < 0.01, *p* < 0.001.

Functional enrichment analysis revealed that EZH2-associated genes were predominantly implicated in crucial cellular processes, including cell cycle regulation, DNA replication, the Fanconi anemia repair pathway, and spliceosome function (Supplementary Fig. 3b). Notably, circadian clock genes such as *PER2* have been shown to directly or indirectly modulate pivotal cell cycle regulators, including *p53*, *p21*, *Cyclin D1*,* CDK1*, and *Cyclin B1*, suggesting a potential interplay with circadian rhythm control mechanisms^15,16^. Figure 6a and b illustrate a significant positive correlation between *EZH2* expression and *CLOCK* (*r* = 0.316, *p* < 0.001) and *CRY1* (*r* = 0.440, *p* < 0.001) expression, as depicted in the scatter plot. In our study, we compared the expression levels of *EZH2* in the normal liver cell line MIHA with those in the HCC cell lines Huh7 and JHH7. The results indicated that the expression levels of *EZH2* in HCC cell lines were significantly higher than those observed in the normal liver cell line MIHA (Fig. 6c, d). We knocked down *EZH2* in JHH7 and Huh7 cells using two specific siRNAs to validate this correlation, and the effectiveness of the knockdown was confirmed by quantitative polymerase chain reaction (PCR) and western blot analysis. Experimental results indicate that the knockdown of *EZH2* leads to the decreased expression of *CLOCK* and *CRY1* (Fig. 6e–h).

### EZH2 knockdown inhibits HCC cell proliferation and induces DNA damage

**Fig. 7:**
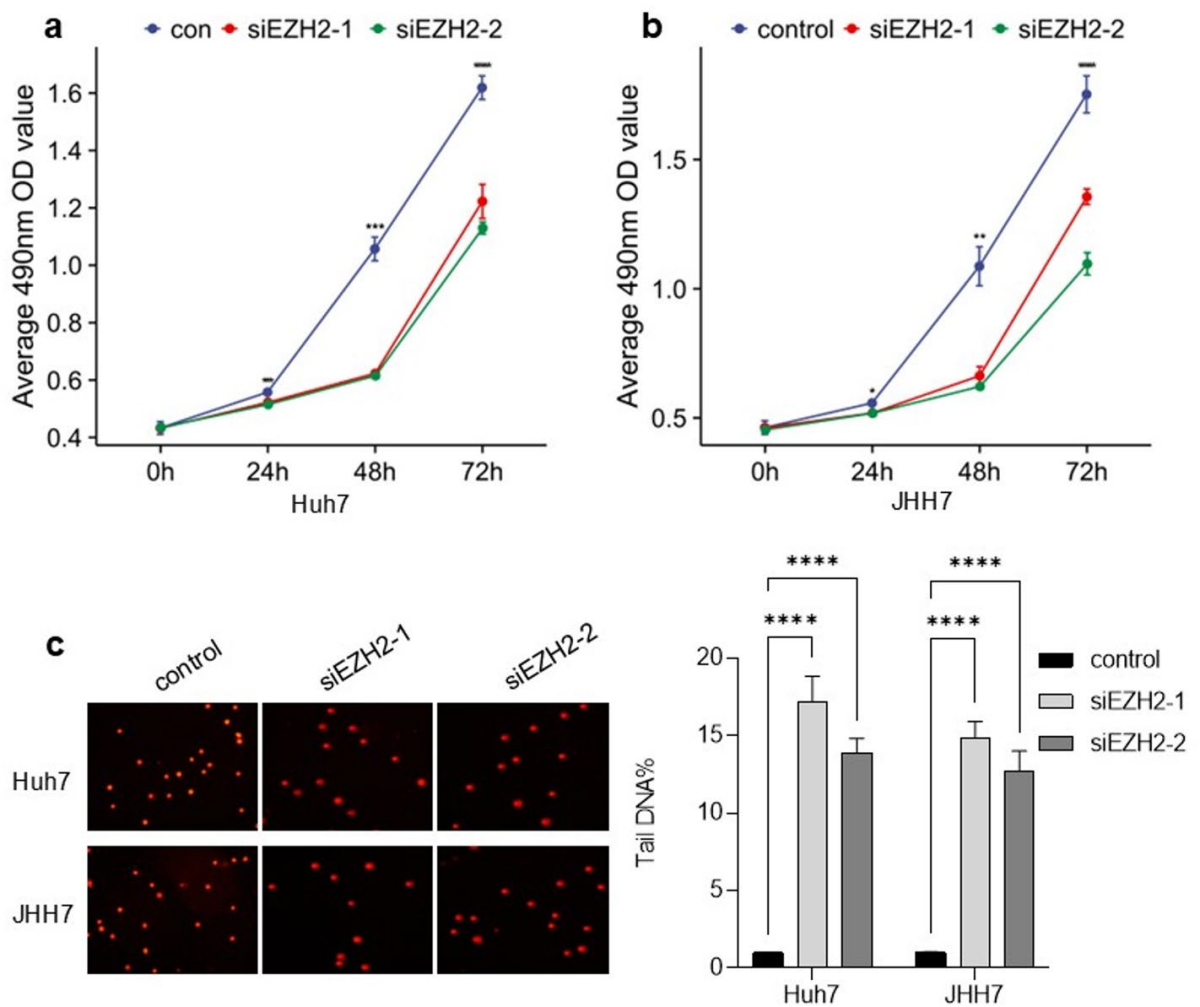
*EZH2* knockdown inhibits HCC cell proliferation and induces DNA damage. Huh7 and JHH7 cells were transfected with siRNA targeting *EZH2*. (**a**, **b**) Cell proliferation was assessed using the CCK-8 assay. (**c**) DNA damage was evaluated using the comet assays. *p* < 0.05, *p* < 0.01, *p* < 0.001.

To investigate the effect of *EZH2* on cell proliferation, we used two specific siRNAs to knock down *EZH2* expression in Huh7 and JHH7 cell lines. Cell Counting Kit-8 (CCK-8) assay results showed that cells with *EZH2* knockdown exhibited a significantly slower proliferation rate compared with control cells (Fig. 7a, b). This observation suggests that the *EZH2* gene may promote cell proliferation (Fig. 7a, b). In addition, the comet assay results showed that cells with *EZH2* knockdown had longer comet tails than control cells (Fig. 7c). Our findings indicate that *EZH2* knockdown causes DNA damage and reduces DNA repair capacity.

## Discussion

HCC continues to be a predominant contributor to cancer-related deaths globally, highlighting the urgent need to discover reliable biomarkers that facilitate early detection and prognosis. This study highlighted the significant overexpression of *EZH2* in HCC tissues and its strong correlation with patient outcomes, underscoring its potential as a critical prognostic biomarker. Unlike previous studies that primarily focused on individual gene expression and exosomal micro-RNAs^17,18^, our research integrated circadian rhythm-related genes, providing a novel perspective on the multifaceted role of *EZH2* in HCC progression.

Circadian rhythms influence the initiation and progression of HCC by regulating multiple processes, including the cell cycle, metabolism, DNA repair, immune microenvironment, epigenetic modifications, oxidative stress, and tumor microenvironment. Dysregulation of circadian rhythm genes—such as *CLOCK*, *BMAL1*, *PER*, and *CRY*—may result in abnormal proliferation, metabolic reprogramming, genomic instability, immune evasion, and microenvironmental remodeling in HCC cells, thereby promoting tumor progression^19–23^. To our knowledge, this study is the first to elucidate the regulatory relationship between *EZH2* and circadian rhythm-related genes (*CLOCK* and *CRY1*) in HCC. Functional enrichment analysis revealed that genes associated with *EZH2* were markedly enriched in pathways related to the cell cycle, DNA replication, and Fanconi anemia repair, all of which strongly connect with regulating circadian rhythms. Experimental validation demonstrated that knockdown of *EZH2* resulted in downregulation of *CLOCK* and *CRY1*, suggesting that *EZH2* may influence HCC progression by modulating these core circadian rhythm genes. Both *CLOCK* and *CRY1* play critical roles in maintaining the cell cycle and DNA repair^5,8^, and their dysregulation may contribute to genomic instability and tumorigenesis. Therefore, *EZH2* may indirectly affect the proliferative capacity and DNA damage repair mechanisms of HCC cells by regulating the circadian rhythm-related genes.

*EZH2* is a core component of the *PRC2* complex. It promotes the proliferation, invasion, and epithelial-mesenchymal transition (EMT) of HCC by catalyzing the trimethylation of histone H3 at lysine 27 (H3K27me3) to silence tumor suppressor genes, such as *CDKN2A* and *PTEN*^10^. *EZH2* exhibits elevated expression levels in HCC, which is notably linked to advanced disease stages, vascular invasion, and unfavorable prognostic outcomes^10^, and regulates the HOXA-AS2/miRNA-ceRNA network to enhance pro-tumorigenic signaling and suppress anti-tumor immunity by reducing CD8^+^ T cell infiltration^10^.

Our analysis revealed that elevated *EZH2* expression was correlated with advanced tumor stages, poor histological grades, and reduced OS rates among patients with HCC. Furthermore, a prognostic model incorporating *EZH2* expression and clinical parameters demonstrated predictive power for patient survival. These findings reinforce the necessity of *EZH2* as an HCC biomarker and suggest its involvement in intricate regulatory networks influencing tumor behavior and patient prognosis. These results warrant further investigation into the mechanistic role of *EZH2* in HCC and its potential as a therapeutic target.

This study revealed a significant negative correlation between *EZH2* expression and OS in patients with HCC, suggesting that *EZH2* may play a critical role in the progression of HCC. This finding aligns with previous research, such as a study utilizing TCGA data, which reported that high *EZH2* expression was associated with poor prognosis in patients with HCC (*n* = 243) and was correlated with advanced tumor stages and increased recurrence rates^24^. Another investigation involving 66 patients with HCC demonstrated that elevated *EZH2* levels were associated with unfavorable clinical outcomes, reinforcing the notion that *EZH2* is an independent prognostic marker for HCC^25^. These studies support the hypothesis that *EZH2* contributes to HCC malignancy through mechanisms such as epigenetic regulation and tumor microenvironment modulation. However, some studies have reported conflicting results. For instance, a study analyzing 379 HCC samples found no significant association between *EZH2* mutations and OS, suggesting that genetic alterations in *EZH2* may not directly influence patient survival^10^. This discrepancy may stem from differences in sample sizes, methodologies, or specific patient populations studied, highlighting the complexity of *EZH2*’s role in HCC. The developed nomogram model, which incorporated *EZH2* expression levels and clinical staging, demonstrated significant clinical utility. Overall, consistent findings across multiple studies underscore the potential of *EZH2* as a therapeutic target and prognostic biomarker in HCC while emphasizing the need for further research to clarify its mechanistic pathways and interactions within the tumor microenvironment.

This study identified various mutation types of the *EZH2* gene in HCC, including missense mutations, truncating mutations, and amplifications. However, the mutation frequency was low (2.9%) and showed no significant correlation with patient survival. DNA methylation levels in the *EZH2* promoter region were significantly elevated in HCC tissues, and the methylation status of certain CpG sites was associated with patient prognosis. These findings suggest that *EZH2* expression in HCC is primarily regulated by epigenetic mechanisms rather than genetic mutations. Future research could further explore the regulatory mechanisms underlying *EZH2* methylation in HCC and evaluate its potential as a therapeutic target. In particular, efforts should focus on identifying key transcription factors that regulate the expression of *EZH2* in specific cellular subtypes. Understanding the functional roles of these transcription factors may reveal novel therapeutic targets. Investigating how these transcription factors modulate *EZH2* activity and associated cellular pathways is crucial for developing targeted strategies to overcome treatment resistance in HCC.

However, this study has several limitations that must be acknowledged. First, a significant limitation of this study is the lack of a specific in vivo model to validate the functional role of EZH2 in the progression of HCC. Moreover, the small sample size may limit the generalizability of our results, and the lack of clinical validation emphasizes the need for larger multicenter studies. Furthermore, the potential batch effects arising from the use of multiple datasets could introduce variability into the expression analysis, complicating the interpretation of the results.

In conclusion, our investigation revealed that increased *EZH2* expression correlates with greater malignancy and dismal prognosis in patients with HCC, underscoring its potential as a prognostic biomarker and therapeutic target. The machine learning approaches employed in this study provided a robust framework for identifying key molecular players in HCC, laying the groundwork for future studies aimed at to elucidating the underlying mechanisms of *EZH2* in tumor progression. Ultimately, these findings may contribute to developing novel therapeutic strategies that could improve patient outcomes in HCC, emphasizing the importance of further investigations into the role of *EZH2* in liver cancer biology.

## Materials and methods

All data were obtained from publicly available databases (TCGA, GEO) and complied with relevant ethical guidelines.

### Screening for core genes between DEGs and circadian rhythm-related genes

RNA sequencing data and relevant clinical details for patients diagnosed with HCC were obtained from TCGA (https://cancergenome.nih.gov/) and GEO (https://www.ncbi.nlm.nih.gov/) databases. Genes associated with circadian rhythms were sourced from the MSigDB (https://www.gsea-msigdb.org/gsea/msigdb/), as presented in Supplementary Tables 1^26,27^. Differential expression profiles (HTSeq-Counts) between the HCC cohort and the control group were analyzed to identify DEGs using the Wilcoxon rank-sum test in R, specifically with the DESeq2 (version 1.36.0) and edgeR (version 3.38.2) packages. Threshold values for identifying DEGs were established as |log2Fold Change| > 1.5 and an adjusted p-value of < 0.01^28,29^. The “ggplot2” package was used to generate mRNA volcano plots.

Venn overlap analysis was used to illustrate the relationship between DEGs and circadian rhythm-associated genes. The Search Tool for the Retrieval of Interacting Genes (STRING; http://string-db.org; version 10.0) was used to predict protein–protein interactions network for *EZH2* co-expressed genes and analyze functional protein associations^30^. Statistical significance was set at a combined score exceeding 0.4, which was deemed statistically significant for interactions. We identified *EZH2* as the central gene using Cystoscape (version 3.10.0).

In addition, this approach involved a preliminary fitting process in which gene expression data were analyzed alongside survival duration using the LASSO regression technique. This was followed by a rigorous cross-validation procedure using the “cv.glmnet” function to ensure the model’s reliability and accuracy. The “coef” function was then used to extract and compute the weights of the selected genes, providing a clearer understanding of their contribution to the analysis.

Finally, patients with complete clinical data from the TCGA database were selected for univariate and multivariate Cox regression analyses to identify candidate genes that serve as independent prognostic risk factors.

### Correlation of gene expression levels with circadian rhythm-associated genes in HCC

The relationship between gene expression levels and circadian rhythm-associated genes such as *CLOCK*, *ARNTL*, *PER1*, *CRY1*, *RORA*, and *NR1D1* was examined using the R statistical programming language. Correlation analysis between these genes was performed using an appropriate R package.

### Expression and prognostic significance of EZH2 in HCC

The TCGA and GEO databases were used to assess the relationship between EZH2 mRNA expression and survival outcomes in patients with HCC and varying clinical characteristics. The UALCAN database was used to evaluate the association between EZH2 expression and patient survival. The R package “survival” (version 3.6) was used to generate OS, DSS, and PFI survival plots based on *EZH2* expression in patients from TCGA database, as well as OS plots from the GEO database. The patients were divided into high- and low-expression groups based on the median expression level of *EZH2*.

### Immunohistochemical staining

Immunohistochemical staining images of normal liver and HCC tissues were obtained from the HPA database (https://www.proteinatlas.org/) to evaluate the varying expression levels of *EZH2* in healthy and tumor tissues^31^.

### Gene ontology and Kyoto encyclopedia of genes and genomes enrichment analysis

Correlation analyses were performed between EZH2 and other mRNAs in HCC using data from TCGA to understand the biological mechanisms and pathways related to *EZH2*. GOKEGG pathway enrichment analyses were performed on genes that demonstrated significant co-expression using the clusterProfiler package in R. Following this, a prognostic and diagnostic nomogram was constructed and validated.

We constructed a nomogram incorporating independent prognostic variables identified through Cox regression analysis to develop a prognostic model for forecasting OS in HCC. These variables include the expression level of *EZH2*, T stage, M stage, and pathological stage. DCA and calibration curves were used to validate the nomogram’s precision and dependability. Furthermore, independent prognostic factors were established, and ROC curves were generated using the “timeROC” software package in R^32^.

### EZH2-associated gene enrichment analysis

In examining cohorts distinguished by high- and low- *EZH2* expression, the 30 genes that exhibited the most pronounced differential expression were further analyzed based on their adjusted p-values. Heatmaps were generated to illustrate the associations between these genes and *EZH2* expression. Functional characterization of the DEGs was performed using GO and KEGG enrichment analyses.

### Gene set variation analysis

The h.all.v2024.1. Hs.symbols.gmt gene set was obtained from MSigDB and used to perform GSVA on *EZH2* expression in HCC samples from TCGA-Liver Hepatocellular Carcinoma (TCGA-LIHC) dataset. Functional enrichment differences were assessed between high- and low-risk cohorts. The threshold for GSVA screening was set at an adjusted p-value of < 0.05, using the Benjamini–Hochberg (BH) method for multiple testing correction. The “GSVA” package was used for the analysis, and results were visualized using the “ggplot2” package.

### Immune cell infiltration level and correlation analysis

ssGSEA and CIBERSORT were used to assess the relationship between various immune cell types and the core gene^33,34^.

### Gene mutations and DNA methylation

This study examined the mutations and CNVs linked to *EZH2* using cBioPortal, emphasizing the correlation between *EZH2* genetic alterations and HCC prognosis^35^. The methylation status of the *EZH2* promoter was assessed using UALCAN^36^, and MethSurv was used to analyze the prognostic significance of the methylation levels of *EZH2*^37^.

### Cell lines and culture

MIHA, Huh7, and JHH7 cells were kindly provided by Dr. Wei Chen. All cell lines were maintained in Dulbecco’s Modified Eagle’s medium (Hyclone, Logan, UT, USA) supplemented with 10% fetal bovine serum (FBS; Hyclone) and 1% penicillin–streptomycin. The cultures were kept in a humidified incubator set at 37 °C with an atmosphere of 5% CO_2_. Each cell line was confirmed to be free of Mycoplasma contamination, and authentication was conducted through genetic profiling of polymorphic short tandem repeat (STR) loci for cultures that had been passaged for more than 6 months after their arrival at our laboratory.

### Western blotting

Cell lysates were prepared in radioimmunoassay buffer (Solarbio, Beijing, China) supplemented with a protease inhibitor cocktail (Roche, Basel, Switzerland). An equivalent quantity of total protein (30 µg) was analyzed via 8% sodium dodecyl sulfate-polyacrylamide gel electrophoresis (SDS-PAGE) and subsequently transferred onto a polyvinylidene fluoride (PVDF) membrane (Millipore, Danvers, MA, USA) by applying a current of 300 mA for 90 min. After a blocking step with 5% skimmed milk at 25 °C for 1 h, the membranes were subsequently incubated overnight at 4 °C with primary antibodies against EZH2 (#5246; 1:1000; Cell Signaling Technology, Danvers, MA, USA), *CLOCK* (#5157; 1:1000; Cell Signaling Technology, Danvers, MA, USA), *CRY1* (13474-1-AP; 1:1000; Proteintech Group, Inc, Chicago, USA), and *GAPDH* (60004-1-Ig; 1:50000; Proteintech Group, Inc, Chicago, USA). Subsequently, the samples were incubated with secondary antibodies conjugated to horseradish peroxidase, and detection was performed using the ECL Plus reagent (GE Healthcare, Chicago, IL, USA).

### SiRNA transfection

The sequences of the si_*EZH2*_ vectors were 5′-GTGCCCTTGTGTGATAGCACAA-3′ and 5′-GGCACTTTCATTGAAGAACTAA-3′. The sequence for the non-target control vector was 5′-TTCTCCGAACGTGTCACGT-3′. The siRNAs were procured from Gema Gene (Shanghai, China). Cells were transfected with si_*EZH2*_ employing Lipofectamine 2000 (Thermo Fisher Scientific Inc. Waltham, MA, USA) according to the manufacturer’s instructions.

### Real-time reverse transcription-polymerase chain reaction

Total RNA was isolated using TRIzol reagent (Life Technologies, Carlsbad, CA, USA) and treated with RNase-free DNase I (Promega, Madison, WI, USA) for 30 min. Reverse transcription was performed using an M-MLV reverse transcription kit (Promega). SYBR Green real-time reverse transcription PCR was performed using the ABI PRISM 7300 Sequence Detection System (Life Technologies). The primer sequences employed were as follows: *CLOCK*: 5′-TCTCAGACCCTTCCTCAACA-3′ (forward) and 5′-TGACCTTCTTTGCACCATCTT-3′ (reverse); *CRY1*: 5′-CTGCGTCTACATCCTGGACC-3′ (forward) and 5′-GAAGCAAAAATCGCCACCTGT-3′ (reverse). Gene expression analysis was performed using the 2 ^−∆∆Ct^ method. The ΔCt value was calculated by subtracting the Ct (threshold cycle) of *GAPDH* from that of the target gene, whereas the ΔΔCt was calculated by taking the difference between the ΔCt value of the experimental sample and that of the control sample. Fold-change was expressed as 2 ^−ΔΔCt, with the relative expression level of the control group normalized to 1.

### Comet assay

Cells were transfected with siRNA, and after incubation for 48 h, they were harvested for further analysis. Following this, comet assays were conducted according to established protocols^38^, and the resulting comet images were visualized using a fluorescence microscope (Leica DMI3000 B; Leica, Wetzlar, Germany). Image analysis was conducted using the Comet Assay Software Project (CASP; Wrocław, Poland) to ensure precise evaluation of DNA damage.

### Cell proliferation

Cells were seeded in 96-well plates at a density of 2 × 10^3^ cells/well, 24 h post-transfection. Cell proliferation was evaluated using the CCK-8 assay (MedChemExpress, Monmouth Junction, NJ, USA) following the manufacturer’s instructions. Absorbance was measured at 490 nm using a multifunctional microplate reader (Thermo Fisher Scientific, Waltham, MA, USA).

### Statistics analysis

Data analysis and visualization were conducted using the R software (version 4.3.2). Box lines and scatter plots were used to assess the expression levels of the *EZH2* gene in patients diagnosed with HCC. The threshold value of *EZH2* expression was determined using the median approach for gene expression. Spearman’s correlation analysis was used to investigate the extent of the correlation between *EZH2*-associated genes and *EZH2* expression. Univariate and multivariate Cox regression analyses were used to identify potential prognostic factors. ROC analysis, along with the commonly utilized method for binary evaluation, was conducted using the *pROC* package to evaluate the ability of *EZH2* expression to distinguish HCC samples from their normal counterparts. The calculated AUC values, ranging from 0.1 to 0.5, reflected discriminative power between 50% and 100%. In all analyses, * indicates *p* < 0.05, which denotes statistical significance; ** indicates *p* < 0.01, signifying high statistical significance; and *** indicates *p* < 0.001, representing extremely high statistical significance.

## Supplementary Information

Below is the link to the electronic supplementary material.

**Supplementary Fig. 1**. Flow chat of the study. **Supplementary Fig. 2**. Expression levels in tumor tissues across clinical subgroups in the TCGA database. **(a)** Age, **(b)** Gender, **(c)** N stage, **(d)** T stage, **(e)** M stage, **(f)** Pathologic stage, **(g)** Residual tumor status, **(h)** Histologic grade, **(i)** OS events. *p* < 0.05, *p* < 0.01, *p* < 0.001; ns: no signification. **Supplementary Fig. 3**. Functional clustering and interaction network analyses of *EZH2*-related genes. **(a)** Heatmap of the top 30 genes positively correlated with *EZH2* expression in HCC. **(b)** Enrichment analyses for BP, CC, MF, and KEGG of *EZH2* co-expressed genes. **Supplementary Fig. 4**. GSVA of differentially enriched pathways between the high and low *EZH2* expression groups. (**a)** Heatmap of significantly enriched pathways. **(b)** Bar plot showing pathway enrichment scores. **Supplementary Fig. 5**. Correlation between*EZH2* expression and immune cell infiltration. **(a-b)** Bubble plots showing correlations between *EZH2* expression and immune cell abundance using the ssGSEA and CIBERSORT algorithms, respectively. **(c-d)** Immune infiltration levels of different immune cells in high and low *EZH2* expression groups, analyzed using the ssGSEA and CIBERSORT algorithms, respectively

Supplementary Table 1. Genes associated with circadian rhythms were sourced from the MSigDB.

## Data Availability

The data supporting the findings of this study are available from the corresponding author upon reasonable request. Public datasets are available from TCGA ( [https://cancergenome.nih.gov/](https:/cancergenome.nih.gov) ) and GEO (https://www.ncbi.nlm.nih.gov/geo/).
