# Supplementary figures and images for: EZH2 expression in hepatocellular carcinoma and its relationship with circadian rhythm-related genes
Source: Sci Rep. 2025 Nov 26;15:42177. doi: 10.1038/s41598-025-26175-x (PMC12658187; doi:10.1038/s41598-025-26175-x)

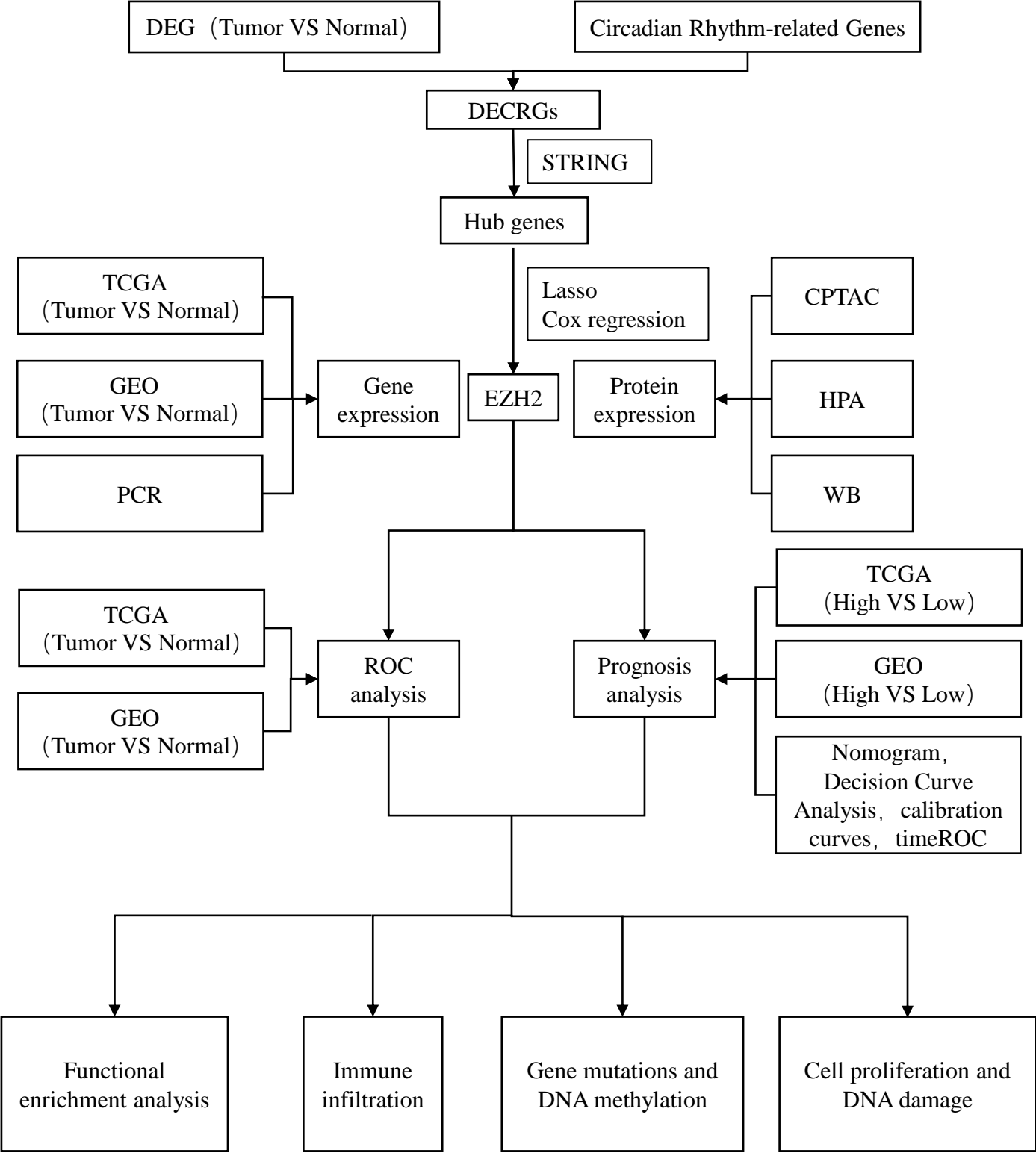

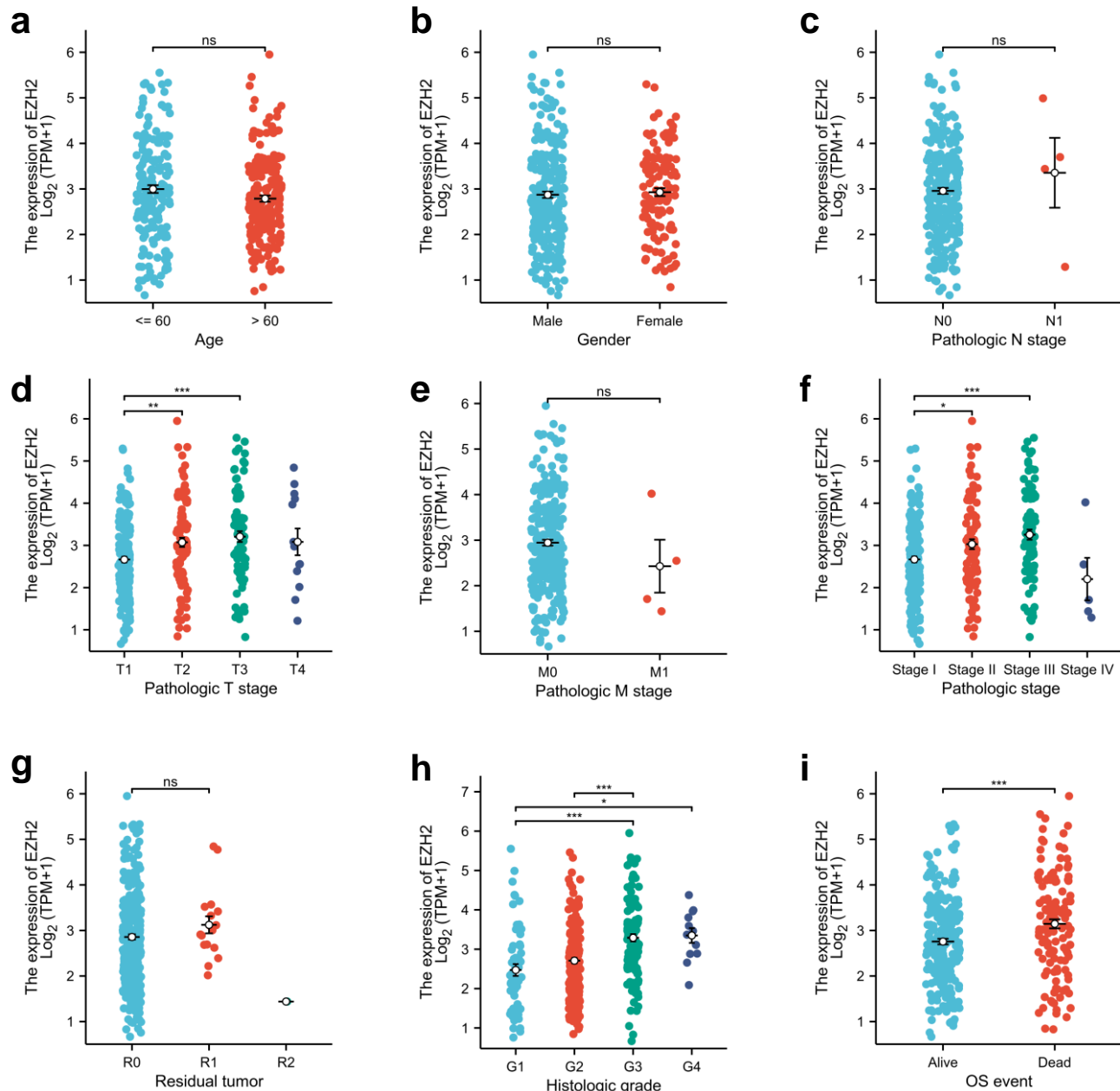

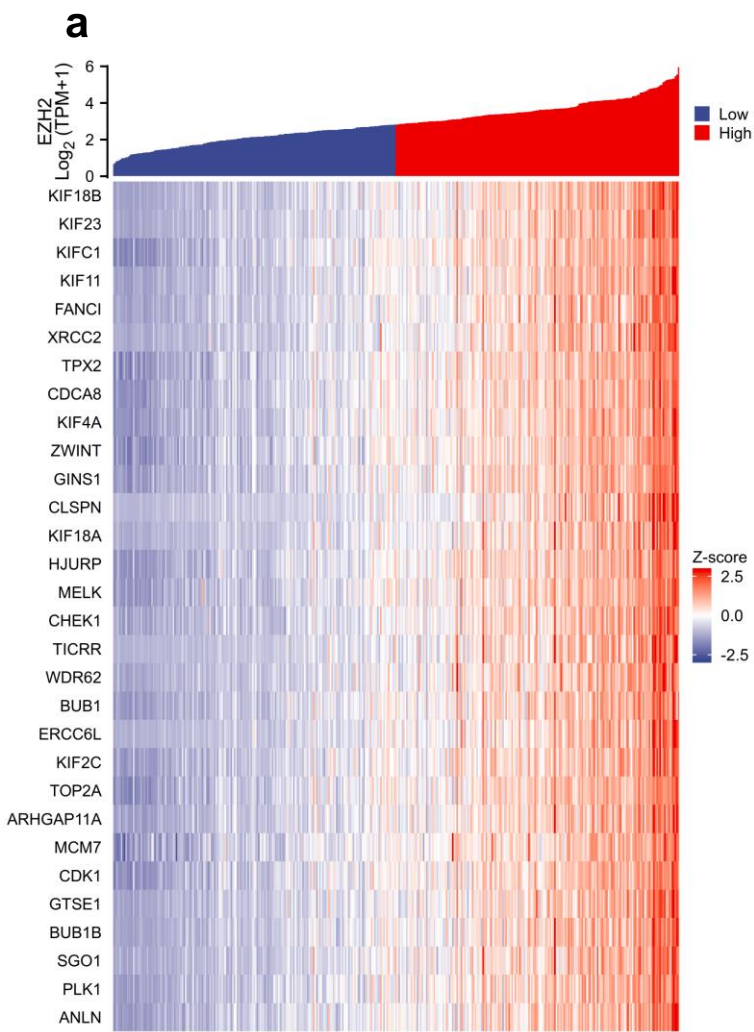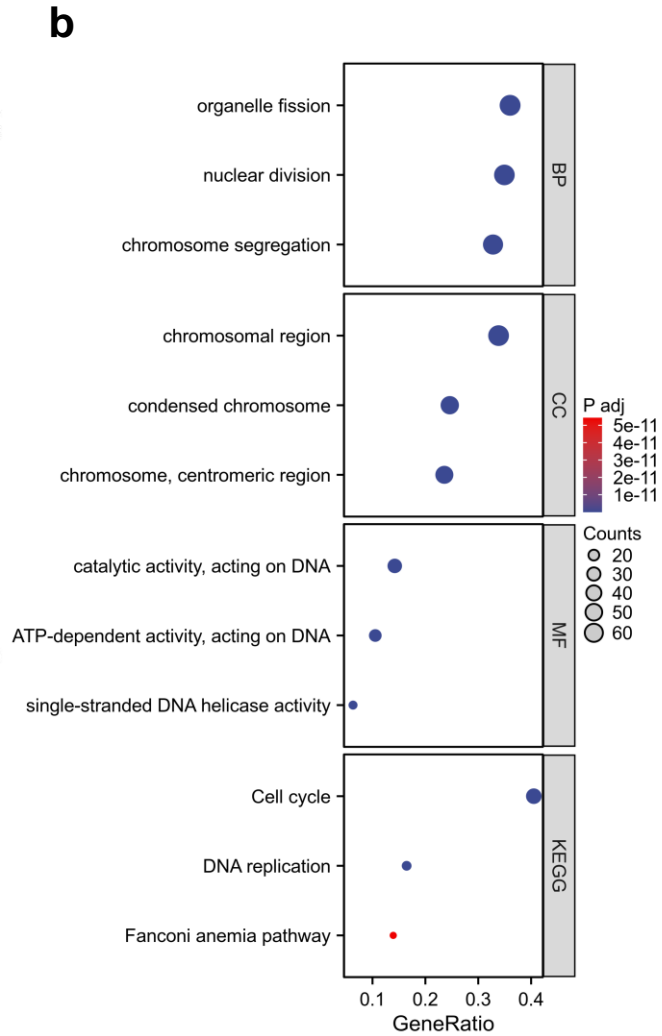

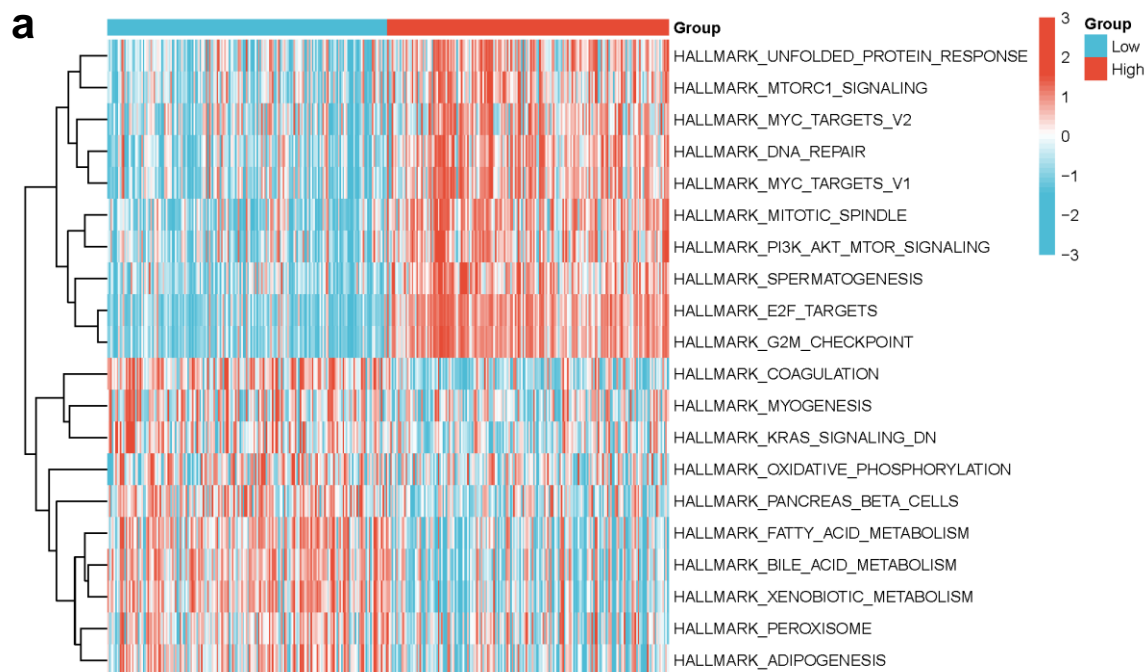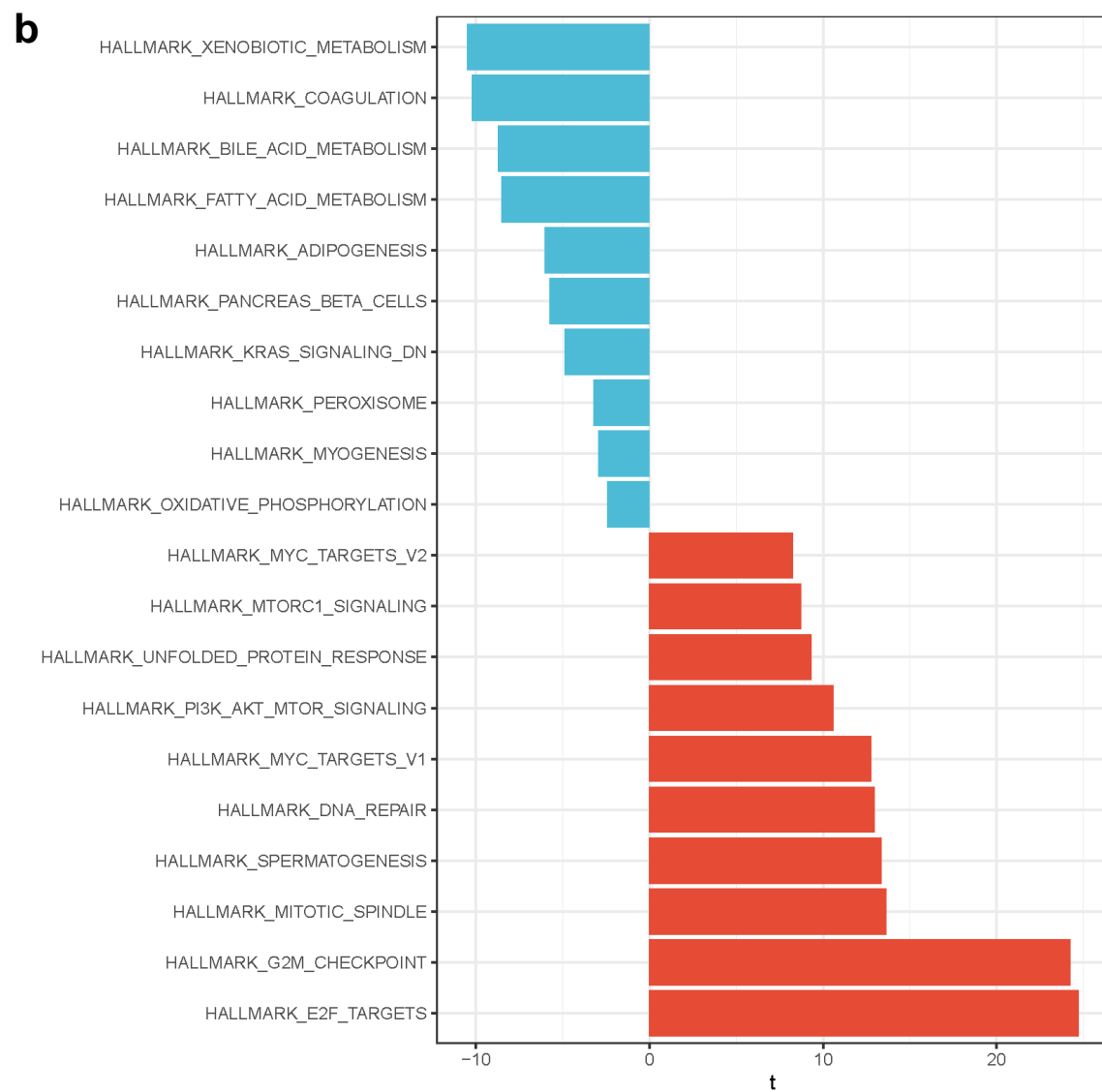

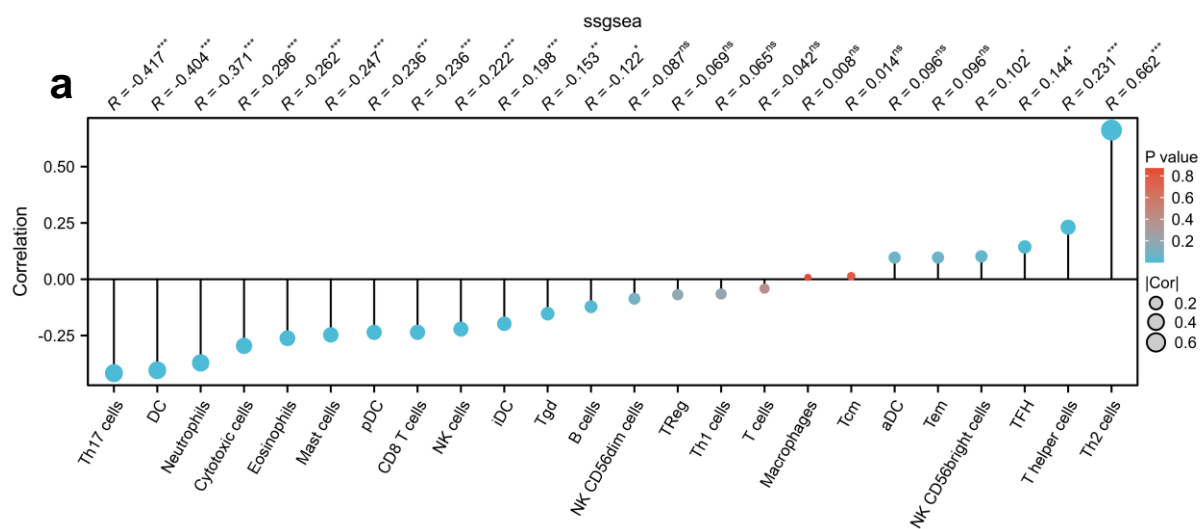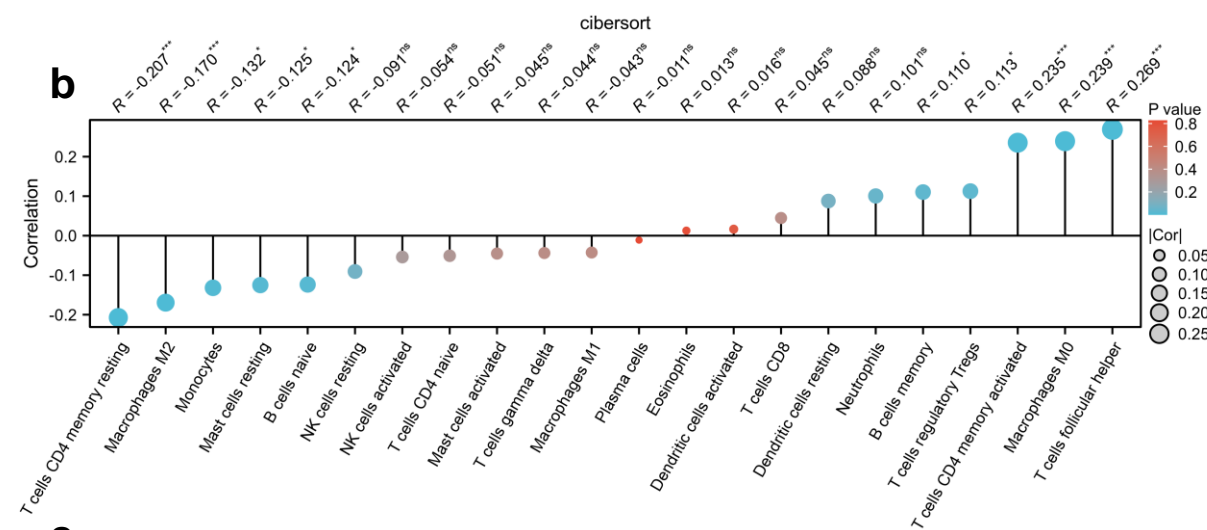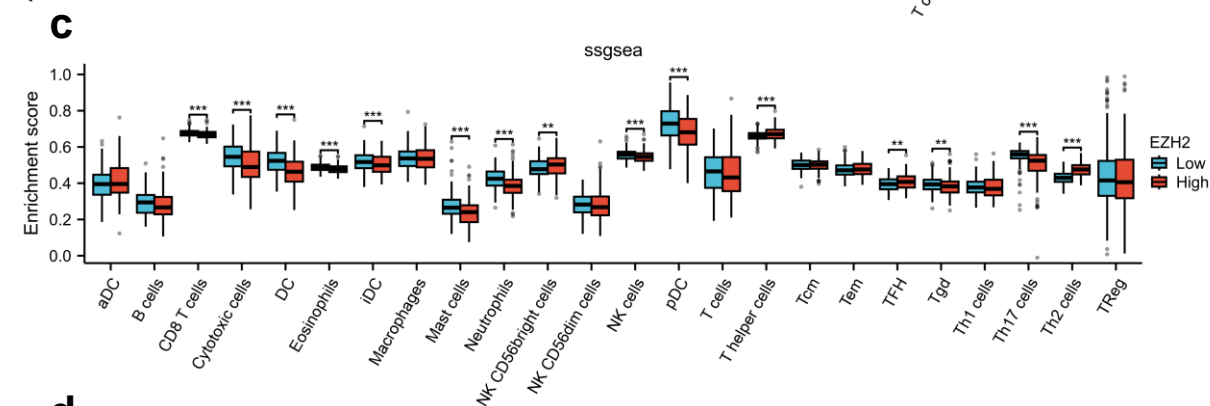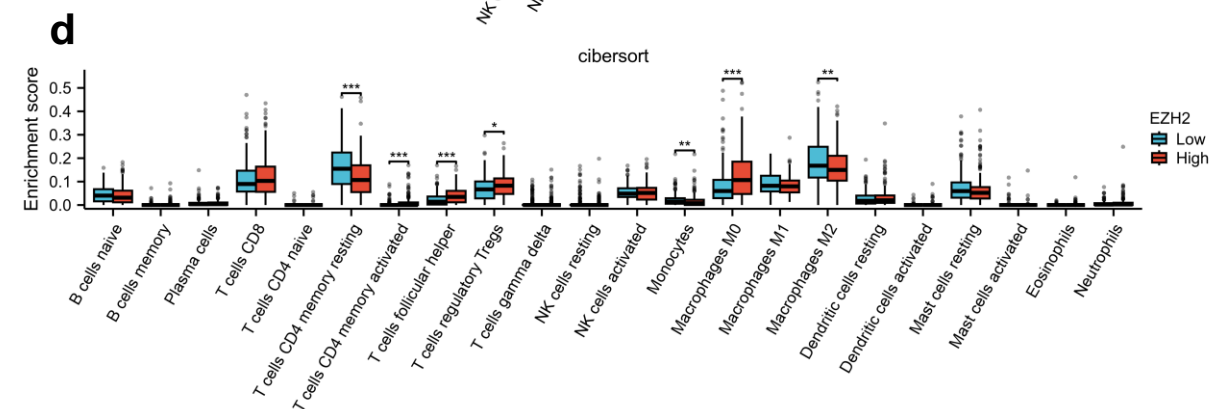

Supplement: Supplementary file 1 — Supplementary Fig. 1. Flow chat of the study. Supplementary Fig. 2. Expression levels in tumor tissues across clinical subgroups in the TCGA database. (a) Age, (b) Gender, (c) N stage, (d) T stage, (e) M stage, (f) Pathologic stage, (g) Residual tumor status, (h) Histologic grade, (i) OS events. p < 0.05, p < 0.01, p < 0.001; ns: no signification. Supplementary Fig. 3. Functional clustering and interaction network analyses of EZH2-related genes. (a) Heatmap of the top 30 genes positively correlated with EZH2 expression in HCC. (b) Enrichment analyses for BP, CC, MF, and KEGG of EZH2 co-expressed genes. Supplementary Fig. 4. GSVA of differentially enriched pathways between the high and low EZH2 expression groups. (a) Heatmap of significantly enriched pathways. (b) Bar plot showing pathway enrichment scores. Supplementary Fig. 5. Correlation betweenEZH2 expression and immune cell infiltration. (a-b) Bubble plots showing correlations between EZH2 expression and immune cell abundance using the ssGSEA and CIBERSORT algorithms, respectively. (c-d) Immune infiltration levels of different immune cells in high and low EZH2 expression groups, analyzed using the ssGSEA and CIBERSORT algorithms, respectively [file 41598_2025_26175_MOESM1_ESM.pdf]
