# Supplementary material for: EZH2 expression in hepatocellular carcinoma and its relationship with circadian rhythm-related genes
Source: Sci Rep. 2025 Nov 26;15:42177. doi: 10.1038/s41598-025-26175-x (PMC12658187; doi:10.1038/s41598-025-26175-x)
Supplement: Supplementary file 2 — Supplementary Table 1. Genes associated with circadian rhythms were sourced from the MSigDB. [file 41598_2025_26175_MOESM2_ESM.docx]

| CRY1 | CSNK1E | MTNR1B | SUV39H1 |
| --- | --- | --- | --- |
| CRY2 | CSNK1D | ADORA2A | SUV39H2 |
| PER2 | PPARGC1A | NLGN1 | CIPC |
| PER1 | HDAC3 | GHRH | NR1H3 |
| ARNTL | NR1D2 | NPS | ADCY1 |
| CLOCK | PER3 | ADORA1 | DRD4 |
| ARNTL2 | NR2F6 | DRD2 | EZH2 |
| BHLHE40 | ATOH7 | CHRNB2 | OPN3 |
| SIK1 | PHLPP1 | DRD3 | GSK3B |
| CRTC1 | GNA11 | HCRTR2 | HNRNPD |
| FBXL3 | GNAQ | PTGDS | ZFHX3 |
| PPP1CA | ID2 | FBXW11 | OPRL1 |
| PPP1CB | PML | RORC | MAGEL2 |
| PPP1CC | TP53 | NKX2-1 | PPARG |
| RBM4 | RBM4B | THRAP3 | PSPC1 |
| NOCT | SOX14 | NONO | PRKAA1 |
| PPARA | USP2 | TIMELESS | PRKAA2 |
| NR1D1 | MTA1 | PASD1 | PRKCG |
| RORA | GHRL | SIN3A | PRKDC |
| BTRC | CRH | SFPQ | MAPK8 |
| MAPK9 | CREM | PROKR2 | HDAC2 |
| MAPK10 | EP300 | NMS | HNRNPU |
| PROX1 | SIRT1 | CLDN4 | HTR7 |
| CCAR2 | NFIL3 | CRX | ID3 |
| RORB | RPS27A | CST3 | ID4 |
| KLF10 | SKP1 | CIART | FAS |
| TOP2A | SREBF1 | AANAT | IL6 |
| UBE3A | UBA52 | DDC | JUN |
| OPN4 | UBC | DDX5 | JUND |
| ROCK2 | NRIP1 | DHX9 | KCND2 |
| MAGED1 | CUL1 | AGRP | KCNMA1 |
| NPAS2 | NCOR1 | DRD1 | LEP |
| BHLHE41 | KLF9 | DYRK1A | ARNT |
| NAMPT | BTBD9 | AHCY | MC3R |
| NCOA2 | ADA | EGR1 | KMT2A |
| DBP | CDK4 | AHR | MTNR1A |
| F7 | PRMT5 | EGR3 | MTTP |
| SERPINE1 | MYBBP1A | ATF5 | NAGLU |
| AVP | PROKR1 | SETX | ATF4 |
| RAI1 | UTS2 | GFPT1 | NGFR |
| CPT1A | CAVIN3 | UTS2R | NOS2 |
| CREB1 | CHRM1 | HCRTR1 | NPY2R |
| CREM | TPH2 | HDAC1 | NTRK1 |
| NTRK3 | TYMS | KCNA2 | CREBBP |
| PAX4 | PROK1 | CSF2 | NCOA6 |
| HEBP1 | OGT | ADRB1 | KLF15 |
| LGR4 | TNFRSF11A | KAT5 | MED1 |
| PRF1 | KCNH7 | PIWIL2 | RXRA |
| PRKG2 | ADIPOQ | ATG7 | SMARCD3 |
| METTL3 | HOMER1 | DDB1 | TBL1X |
| PTEN | CARTPT | KDM2A | TBL1XR1 |
| KDM5A | ARNT2 | TARDBP | CHD9 |
| RELB | HS3ST2 | MTOR | HELZ2 |
| PROK2 | OPN5 | HNF4A | NCOA1 |
| SFTPC | FBXL21P | SRRD | TGS1 |
| SIX3 | FBXL6 | SIRT6 | ATF2 |
| SLC6A4 | FBXL22 | FBXW7 | CRTC2 |
| SLC9A3 | PDE6B | SIAH2 | NR3C1 |
| STAR | FBXL12 | USP7 | HIF1A |
| HNF1B | FBXL8 | USP9X | MEF2C |
| TH | FBXL17 | SPSB4 | MEF2D |
| TOP1 | GHRHR | CDK1 | CRTC3 |
| TPH1 | PARP1 | CARM1 | USP46 |
| UBB | ERC2 | PIGF | H2BC15 |
| ELOVL3 | GFRA1 | PPP1R3C | STBD1 |
| TOB1 | GSTM3 | PPP2CB | VAPA |
| TUBB3 | GSTP1 | PSMA4 | QKI |
| CEBPB | HLA-DMA | PURA | HERPUD1 |
| NCKAP1 | DNAJA1 | SUMO3 | DAZAP2 |
| SF3A3 | HSPA8 | BTG1 | WDR5 |
| RBPMS | IDI1 | CLDN5 | CHEK1 |
| CBX3 | MYF6 | SUMO1 | DELEC1 |
| EIF4G2 | G0S2 | UCP3 | ATR |
| ETV6 | AZIN1 | UGP2 | NCOA4 |
| TAB2 | ZFR |  |  |
